# SLAM in Dynamic Environments: A Deep Learning Approach for Moving Object Tracking Using ML-RANSAC Algorithm

**DOI:** 10.3390/s19173699

**Published:** 2019-08-26

**Authors:** Masoud S. Bahraini, Ahmad B. Rad, Mohammad Bozorg

**Affiliations:** 1 Department of Mechanical Engineering, Sirjan University of Technology, Sirjan 78137-33385, Iran; 2 School of Mechatronic Systems Engineering, Simon Fraser University, Surrey, BC V3T 0A3, Canada; 3 Faculty of Mechanical Engineering, Yazd University, Yazd 89195-741, Iran

**Keywords:** autonomous robot, SLAM, DATMO, RANSAC, multi-target tracking, deep learning, R-CNN

## Abstract

The important problem of Simultaneous Localization and Mapping (SLAM) in dynamic environments is less studied than the counterpart problem in static settings. In this paper, we present a solution for the feature-based SLAM problem in dynamic environments. We propose an algorithm that integrates SLAM with multi-target tracking (SLAMMTT) using a robust feature-tracking algorithm for dynamic environments. A novel implementation of RANdomSAmple Consensus (RANSAC) method referred to as multilevel-RANSAC (ML-RANSAC) within the Extended Kalman Filter (EKF) framework is applied for multi-target tracking (MTT). We also apply machine learning to detect features from the input data and to distinguish moving from stationary objects. The data stream from LIDAR and vision sensors are fused in real-time to detect objects and depth information. A practical experiment is designed to verify the performance of the algorithm in a dynamic environment. The unique feature of this algorithm is its ability to maintain tracking of features even when the observations are intermittent whereby many reported algorithms fail in such situations. Experimental validation indicates that the algorithm is able to perform consistent estimates in a fast and robust manner suggesting its feasibility for real-time applications.

## 1. Introduction

Autonomous navigation in unknown and dynamic environments is a central problem in many robotic applications. Much of the reported literature focuses on a subset of this problem, i.e., Simultaneous Localization and Mapping (SLAM) in static environments [1,2]. Readers may refer to survey papers [3,4,5,6,7] that outline recent developments in SLAM. Although such methodologies are effective in static environments, they fail in realistic scenarios whereby moving objects are present. In recent years, however, researchers have attempted to design SLAM algorithms for unstructured, uncertain, and dynamic environments [8,9,10,11]. In order for an autonomous robot to navigate through an unknown environment; two problems need to be resolved: (i) SLAM problem, which builds and updates the environment map while localizing the robot with respect to that map and (ii) Detection And Tracking of Moving Objects (DATMO) nearby the robot and estimating their future behavior. In such states, there may be other autonomous robots, humans, and stationary objects in the scene. DATMO may be improved with a prior knowledge about the class of moving objects. It should also be noted that the problem is more difficult in an outdoor scene, which is inherently more complex. However, the focus of this paper is on indoor settings. 

Although there are many algorithms available for DATMO from 2D and 3D laser sensors or from stereo and monocular cameras, the selection of the best algorithm is not a straightforward problem. If the objective is to detect objects (static or dynamic) in order to achieve a safe motion for the robot and to prevent collision, the fusion of laser and vision data facilitate robust feature detection. The process also alleviates the computational effort of SLAM algorithm implementation as opposed to high computational techniques such as vision only algorithms. Multi sensor fusion techniques are nowadays widely used for autonomous robots. Sensor fusion of the data can be grouped into three different levels: before object detection, after object detection, and after tracking the moving objects [12].

It should be noted that most of SLAM and DATMO algorithms are prone to error in the presence of moving objects at slow speeds. Also, if there are many moving objects in the environment, SLAM algorithms that are based on mapping static objects fail. In some SLAM algorithms for dynamic environments, the assumption of tracking only one target, makes them impossible to implement in a real environment. The grid-based algorithms are also locally applicable and require large amounts of computing and memory in large environments. Also, graph-based algorithms become divergent where there is no static object in the surrounding environment. The proposed multilevel-RANdomSAmple Consensus (ML-RANSAC) algorithm alleviated the main drawbacks of SLAM in presence of moving objects while uses state-of-the-art methods for object detection like deep learning.The suggested algorithm is able to detect moving objects via a trained R-CNN (Region-based Convolutional Neural Networks) to overcome the tracking of the low speed moving objects. Since the proposed method considers both static and dynamic objects in a dynamic environment, it works robustly in an environment in presence of many moving objects. Also, the proposed approach can be implemented in real environment while tracking multiple targets. In addition, a feature-based algorithm is used in the proposed algorithm to be consistent in an environment without any static object. 

In this paper, we apply sensor fusion of 2D laser scanner and vision sensor at the object detection level. The vision data helps the algorithm to find features in dynamic environments. A Convolutional Neural Network (CNN) [13] is trained to learn deep features from the input data and to segment them into the moving and stationary objects. Then, the depths of the features are extracted from the laser range finder. As a result, the problem of SLAM and DATMO are solved concurrently without separating the estimator into two parts. In other words, the stationary as well as dynamic objects are predicted and updated using one estimator. In such cases, we focus on obtaining a consistent map of static objects, discriminating between static and dynamic objects and concurrently estimate and track moving features. It should be noted that the grid-based and point-cloud-based methods for SLAM and DATMO, in general, suffer from high computational cost and are restricted to local mapping. Although the grid-based SLAM methods are well-suited for navigation and obstacle avoidance tasks, they do not provide a higher-level understanding of the environment. Autonomous robots can assist human users by accepting and understanding commands, such as finding objects or places. Therefore, the features of the environment should be included in the built map. Towards these goals, we use a feature-based and semantic mapping in a dynamic environment. 

The contributions of this paper can be summarized as follows: (i) We propose a novel method referred to as ML-RANSAC algorithm in conjunction with machine learning and multi-target tracking (MTT). The algorithm provides a new approach for data association, updates carefully a track in proximity and occlusion situations, and prevents the incorrect initialization of a track and false deletion of a true track. Using the ML-RANSAC algorithm, moving objects can be detected, while localization of the robot and mapping of stationary objects are also in progress. (ii) The algorithm is designed to make hard decisions for data association in situations like proximity and occlusion situations. In the case that data association can only be performed using the compatibility matrix, the RANSAC method are not used, which has usually a high level of computational efforts. It results in executing the algorithm at a very high speed, in dynamic environments. The proposed ML-RANSAC algorithm is suitable for detecting moving objects, even when receiving intermittent observations. (iii) A deep neural network is trained for detection and classification of objects in a dynamic environment. (iv) A practical experiment is conducted to verify the applicability of the algorithm in real and dynamic environments. 

The rest of this paper is organized as follows. Section 2 reviews the related works. Object detection and classification approach using deep learning method is described in Section 3. Section 4 broadens the ML-RANSAC algorithm, including the details of the algorithm. Section 5 provides the experimental results using real data obtained from a dynamic environment. Finally, discussion, conclusion and perspectives are described in Section 6.

## 2. Related Works

Object classification is an important problem in a dynamic environment. A perfect detection and classification of moving objects results in higher accuracy in collision avoidance implementations. As a solution, multiple sensor detections can help the representation of objects and the perception process in order to build functional model of the environment, especially in the presence of moving objects. The fusion of LIDAR and vision is a popular choice in object detection and tracking [14]. Vision provides robust data association for SLAM if features can be detected in images and be matched from different viewpoints. A survey on Visual SLAM algorithms and techniques is reported in [4]. Recent advances in computer vision and machine learning has provided new solutions to object detection and tracking problems. Image classification, object detection, and semantics segmentation help an autonomous robot have a better sense of its surrounding environment. Deep learning as a branch of machine learning offers practical solutions for object detection and classification. It should be noted that most of the classical object detection methods are not suitable for real environments and are prone to errors in unstructured dynamic environments [15,16,17]. The deep learning approach for object detection and classification from RGB-D images using both image and depth features is studied in [18], and especially for robotics application, is investigated in [19], which delivered reasonable results in object detection and classification. Dynamic learning rate strategies and adaptive online model updating technique are proposed in [20] to robustly track objects in vision data. The reported results show that the proposed approach is able to address the problems of occlusion, scale variation, fast motion, and motion blur in real time applications. The final goal of the object detection can be summarized as finding a group of pixels in an image. After extracting the pixel group as an object in the image, the distance of the object from the robot can be measured by a depth sensor. A lowpower and real time deep learning is implemented on Embedded System in [21] for multiple object tracking. The promised results are reported by authors under real challenging scenarios including low light and high contrast conditions, glass reflections, high velocity, and shadows. A 3D multi-object tracking using deep learning detections is investigated in [22]. The reported results show that the proposed algorithm is able to accurately track objects and correctly handle data associations in real-time.Readers may refer to survey papers for recent vision-based and deep learning pedestrian detection methods [23], deep learning methods [24], and their applications for mobile robots and object detection [25].

For object tracking, each measurement output assigned to an existing track might either be mistaken as a new track or is ignored as a false detection. Furthermore, an erroneous data association results in updating a track with wrong measurement, initialization of a false track, or deletion of a real track [26]. The classical data association techniques in SLAM and DATMO area can be summarized into at least three groups: Global Nearest Neighbor (GNN); Joint Probabilistic Data Association (JPDA); and Multiple Hypothesis Tracking (MHT). Each method has its own merits and drawbacks. The nearest neighbor is not robust whereas JPDA requires a priori knowledge about the number of targets to be tracked and MHT is computationally expensive with exponentially growing number of hypotheses (hypothesis tree) and difficulty of implementation and coding [27]. Especially in dynamical environments, MHT is more computationally demanding, and the nearest neighbor algorithm can diverge easily. A main drawback of these methods is that the current step of data association depends on previous steps. For instance, an unexpected change in the direction of an object may lead to losing tracking of the object. The Interacting Multiple Model (IMM) technique was proposed [28] to address this drawback; however, this was achieved at the cost of increasing the computational effort and complexity of the algorithm. RANSAC (RANdomSAmple Consensus) is one of the most successful approaches to obtain the robust estimation from a dataset that contains both inliers and outliers. It is an iterative method to estimate parameters of a model by constructing a model hypothesis from a minimal set of observed data and evaluating how many measurements support that hypothesis. The generated hypotheses are compared to obtain an hypothesis with the highest consensus. The RANSAC method is robust to sudden motion changes, but faces difficulty from random selection of all hypotheses in the current frame step. For a survey on RANSAC techniques, see [29,30]. Also, a survey on recently researched data association techniques for multi-object tracking can be found in [31].

Wolf and Sukhatme [32] divided the problem of SLAM and DATMO in two parts by differentiating between static and dynamic parts and creating maps of dynamic environments based on the static parts using an EKF. However, this method is prone to errors in some situations especially in the presence of slow moving objects. Also, the SLAM algorithms, which are based on static objects mapping, will fail if the majority or all of the features in the environment are dynamic. The pioneering work on SLAM in conjunction with DATMO was reported by Wang et al. [8]. The authors established a mathematical framework to solve the problem of SLAM and moving object tracking by decomposing the estimation problem into two separate posteriors for stationary and moving objects. They validated the algorithm on laser rangefinder data in an urban environment. However, the proposed grid-based algorithm in this work is locally applicable and require high computational efforts in large environments. Migliore et al. [33] investigated segmentation of features, which independently move from a moving camera using a MonoSLAM algorithm along with a bearing-only tracker. They also addressed the convergence problem to obtain an accurate estimate of the moving objects in the scene. The proposed approach might fail in the presence of moving objects at slow speeds and it is not applicable for multiple target tracking. In order to alleviate the computational complexity, the solution for SLAM and tracking were decoupled. Darms et al. [34] investigated a sensor independent algorithm applied to an autonomous vehicle in urban environments to classify and track the dynamic objects. However, the feature extraction algorithm searches for “L” shapes only in laser data which can lead to false detection and classification. Lin et al. [9] studied localization, mapping, and moving object tracking in an indoor environment by both stereo and monocular cameras and showed that the performance of stereo SLAMMOT (Simultaneous Localization And Mapping, Moving Object Tracking) is superior than monocular SLAMMOT in dynamic environments. The limitations of this algorithm were, however, the need for a reliable 2D feature tracking over images to perform stable stereo SLAMMOT and the assumption of a single target tracking in the experimental environment. Baig et al. [12] studied the fusion of laser scanner and stereo-vision at the object detection level for moving objects tracking in an intersection like scenario. Moving objects were detected by comparing new laser measurements with previously constructed grid map, after obtaining the local position of the vehicle and incrementally constructing a local grid map from environment. Detected moving objects were tracked by a MHT algorithm along with an adaptive IMM filter in [10]. The proposed grid-based algorithm in this work is also locally applicable and require high computational efforts in large environments. Azim and Aycard [35] investigated detection, classification, and tracking of moving objects using a 3D range laser in dynamic outdoor environment, where GNN technique was employed for data association.However, the applied data association technique is not proper for dynamic environments and will fail, especially in proximity and occlusion situations. An EKF-based algorithm for multi-robot simultaneous localization and tracking, using MHT, was proposed by [11] to solve data association problem. The advantage of multi sensor fusion and classification at detection level was studied in [36] by using LIDAR, radar, and camera as the main sensors for the moving object detection and tracking. A survey on the problem of visual SLAM and structure from motion in dynamic environments can be found in [37]. A novel approach for SLAM along with multiple moving objects tracking in dynamic environment was proposed by Bahraini et al. [38]. The proposed ML-RANSAC algorithm in that work alleviated the main drawbacks of SLAM in presence of moving objects but it still suffers from the use of classical methods for object detection.Applying the new methods for object detection helps the ML-RANSAC algorithm to work robustly in dynamic environments. The current work improves the prior published work [38] by the authors in the following aspects: (i) 2D laser scanner and vision data are fused in the current work, whereas we used only 2D laser sensor in the previous work, (ii) A R-CNN is trained to detect objects, whereas a classical method has been used in the previous one, (iii) the proposed algorithm is able to classify detected objects to moving and stationary objects by applying the trained R-CNN, whereas the previous work might not be consistent in classifying them since there in no information about the type of objects in laser data, (iv) A new version of ML-RANSAC algorithm is adapted with machine learning in the current work, (v) A new practical experiment is conducted to verify the applicability of the algorithm in real and dynamic environments by fusing laser and vision data.

Implementation of a robust method for object detection and classification improves MTT in a dynamic environment. Data association is another important problem in dynamic environments, especially in proximity and occlusion situations. Tracking multiple targets is also a challenge when targets are moving around each other. It can be concluded that the object classification, reliable data association and certainly a robust tracking algorithm are the main problems of navigation in a dynamic environment. In this paper, we have proposed an efficient algorithm to solve the problem of classification of objects, the association of data, and the tracking of moving objects in a dynamic environment. Deep learning is used to detect objects and to classify them to stationary and moving objects. By applying ML-RANSAC algorithm to observation data, we can track moving objects while we are localizing the robot and mapping stationary objects via EKF.

## 3. Object Detection and Classification by Deep Learning

An overview of moving object detection with laser scanners can be found in [39]. In that paper, it is discussed that there are at least three reasons to use multiple sensors in detection of moving objects. Firstly, applying multiple sensors ensures that all the area is covered. The second reason is to improve the quality of detection by combination of different type of sensors such as laser, radar, and vision. Finally, multiple sensors address the redundancy requirement. 

**Figure 1:**
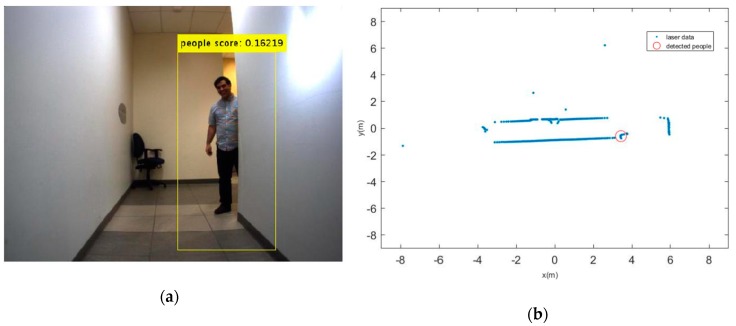
(**a**) Detected moving person; (**b**) His extracted depth from laser sensor.

Reviewing the available literature shows that the perception problem and detection of features in dynamic environment are investigated by using variety of sensors in different scenarios. The present algorithms can be implemented by different set of sensors such as RGBD, monocular camera, stereo camera, laser, and radar, or even by each of these sensors alone. We employ a vision sensor along with laser scanner to detect features and their positions with respect to the robot in a dynamic environment. It should be noted that the use of classical methods for object detection is not robust and it might fail in some situations and environments. For instance, it is difficult to locate the person in Figure 1 form the laser data alone, but by fusing vision sensor and depth sensor we can detect this person and his position.

**Figure 2:**
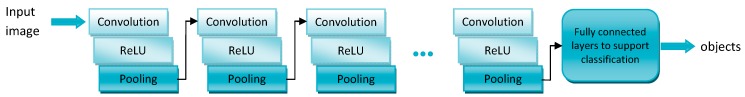
An illustration of the convolutional neural network structure.

Deep learning has been applied to detection and classification of objects with impressive results. The fundamental aspect of deep learning can be found in the classical neural network literatures. One of the popular deep learning architectures is called CNN, which automatically learns a hierarchical feature representation directly from image data. CNN is a special type of feed-forward networks inspired from the behavior of a visual cortex. Many hidden neurons and layers are the core components of a CNN to learn from a dataset comprised of the images and their corresponding labels. CNNs are built up by convolutional layers, Rectified Linear Units (ReLU) and pooling layers, which allow the network to encode certain images properties. The convolutional layers convolve their input with a set of learnable filters/weights. The ReLU layer enables the network to approximate the nonlinear transformation between image pixels and the semantic content of an image. The pooling layers are a form of nonlinear down-sampling to reduce the number of parameters and the duration of computation in the network while consolidate local image features.The implementation for this study is similar to the conventional structure of CNN, which is shown in Figure 2. 

In addition to algorithmic innovations by new training paradigms, the advances in hardware and computing capabilities using Graphics Processing Units (GPUs) can speed up the training process in deep learning. We need an extensive amount of labeled data to train a CNN, which is difficult to achieve for a special object. Additionally, deep learning needs extensive computational resources to perform well. To overcome these drawbacks, R-CNN can be used. R-CNN is a state-of-the-art visual object detection framework, which uses a CNN to classify image regions within an image [40]. Finally, a set of rectangular (group pixels) will be extracted as detected objects from input images of arbitrary size, which a label and confidence are associated with each one. A low accuracy score indicates the low confidence for detected objects. The application of R-CNN to images results in decreasing the computational cost of a CNN by processing only the regions that are likely to have an object instead of classifying all regions, using a sliding window. To improve the performance of a pre-trained CNN, a transfer learning can be used, which retrains the CNN on new data. A pre-trained network, which is trained on a large collection of images, can be trained more for a small number of target images. Then, it can be applied on a wide range of images for detection and classification of objects. Transfer learning or fine-tuning offers a way to solve a new classification or detection task by using a pre-trained CNN. For efficient and easy fine-tuning, small adjustments will be applied to the primary weights of CNN. It can be concluded that transfer learning leads to two main advantages: (i) prevent the training a CNN from base, which is slow, expensive, and tricky, (ii) the number of images required for training will be reduced. 

One of the important landmarks in an indoor environment is “Door”, which provides the entrance and exit points of rooms. It can be used to assist blind people to independently access to unknown environments. A service robot can be able to find places or objects, to access rooms and corridors, and to know where it is, by detecting doors in a domestic environment. Also, doors are used as predefined beacons in different algorithms of SLAM, but detecting doors and their position still presents a challenge of computer vision in an unknown environment. As a prototype, we will apply deep learning algorithm to detect doors and people in the dynamic environment, although the current trained network has also learned for other objects of the environment such as chairs, signs, and mobile robots.

## 4. ML-RANSAC Algorithm for SLAMMTT

SLAM is a fundamental requirement for an autonomous robot navigating in an unknown or partially known environment, when external signals such as global positioning system (GPS) are not available. In the SLAM, the robot typically starts at an unknown initial location, navigates through the unknown environment with a population of landmarks. The robot is equipped with one or more sensors to obtain odometry data and distance of the objects with respect to the robot. One of the common sensors for SLAM is a laser scanner, which identifies the range and bearing of the landmarks. Whilst navigating the environment, the robot builds a complete map of landmarks and uses them to provide estimates of the robot location by a recursive process of prediction and update. The details of a SLAM system can be found in [41,42]. The capabilities of an autonomous robot can be further extended if it can safely move through a dynamic environment. SLAM along with DATMO helps the robot to have a complete knowledge about its surrounding environment. When information from sensor measurements is not accurate and/or in the presence of uncertainty, incompleteness, or conflicting information, data fusion at detection level results in increasing the reliability of detection [36]. ML-RANSAC was originally developed as a recent extension of the RANSAC algorithm by Bahraini et al. [38]. The motivation was to track moving objects while running SLAM.

**Figure 3:**
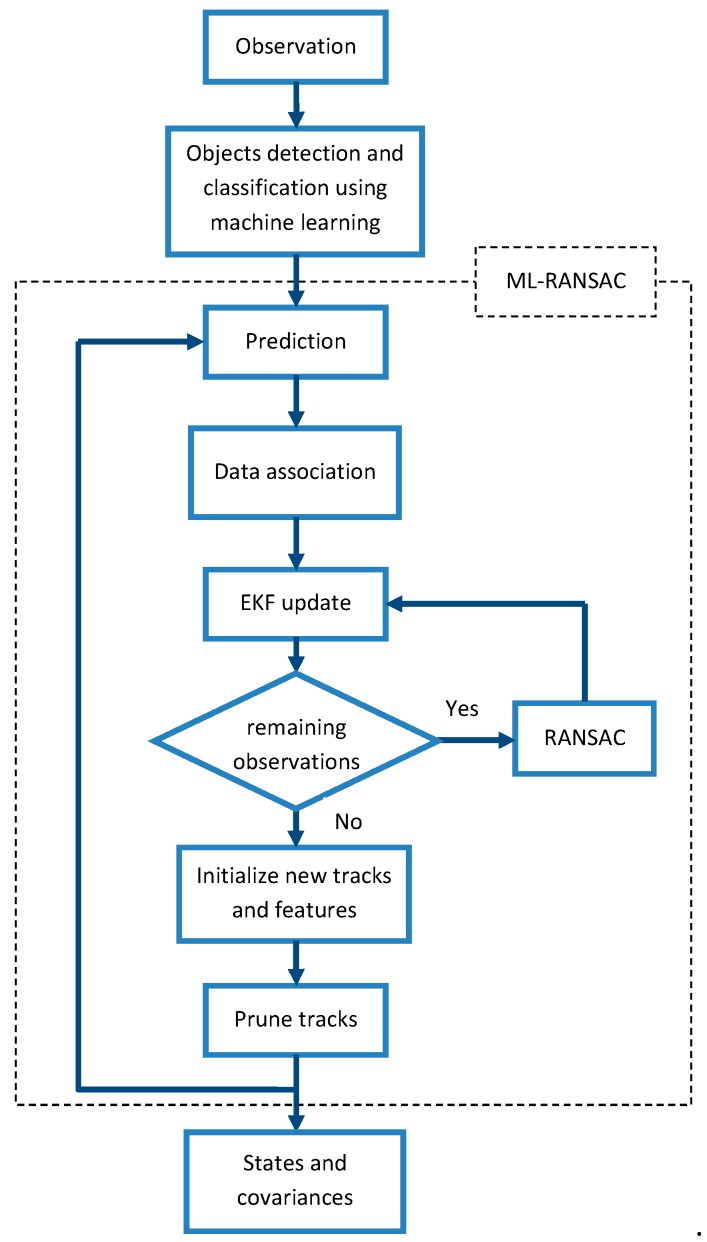
Overall scheme of multilevel-RANdomSAmple Consensus (ML-RANSAC) algorithm for Simultaneous Localization and Mapping with multi-target tracking (SLAMMTT).

The proposed ML-RANSAC algorithm in conjunction with machine learning is described in this section. An overall scheme of the ML-RANSAC algorithm for SLAMMTT is shown in Figure 3. Firstly, we use the received observation data from laser scanner and vision sensor for object detection and classification. A trained R-CNN is utilized to detect objects and classify them to stationary and moving objects. Then, classified objects are conducted to the ML-RANSAC loop, where the prediction step, data association, and measurement update are performed. Furthermore, the expired tracks are pruned and new tracks are initialized. Finally, the location of the robot and the map of the stationary objects along with the list of the tracks and their locations are delivered. Detailed formulation and description of the proposed algorithm are provided in the rest of this section. 

It should be noted that the proposed algorithm is generally designed for any stationary and moving objects, not especially for doors and people, which are used in this paper as stationary and moving objects, respectively. Algorithm 1 summarizes the implementation of the ML-RANSAC. 

**Algorithm 1.** Multilevel-RANSAC.**Require:**$\hat{X}$^+^(*k* − 1), *P^+^*(*k* − 1) (EKF estimated state and covariance at step *k* − 1), *Z*(*k*) (measurement at time step *k*) **Ensure:**
$\hat{X}$^+^(*k*), *P^+^*(*k*) (EKF estimated state and covariance at step *k*), **for** each time step *k*
**do** 2- Prediction(); 3- Individual_compatibility_match(); 4- Compute_ J(); 5- Find the tracks with only one compatibility match in the matrix *J* 6- EKF_update(); 7- **if** there is another observation which needs a decision making **then** a. RANSAC(); b. EKF_update(); 8- **end if** 9- Prune_tracks(); 10- **end for**

**Figure 4:**
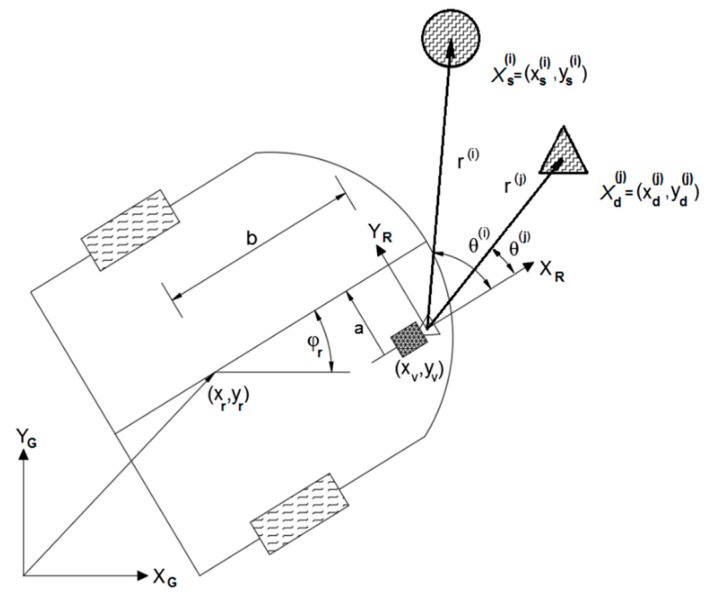
Vehicle and observation kinematics [38].

The algorithm requires EKF estimated state and covariance at previous time step and observation measurement from the current time. On its output, it provides estimated state and covariance at the current time step. The basic layout of the observation process and robot model is presented in Figure 4.

It should be noted that, the state vector of the mobile robot $X_{k}^{r}={\left[\begin{array}{ccc} x_{k}^{r} & y_{k}^{r} & \phi_{k}^{r} \end{array}\right]}^{T}$ has the size of three which is described by its position $\left(x_{k}^{r},y_{k}^{r}\right)$ and orientation $\phi_{k}^{r}$. Also, the state vector of a static object $X_{k}^{s}$ has the size of two, which is described by its position, while the state vector of a moving object $X_{k}^{d}$ has the size of four, which is described by its position and velocity. It is assumed that there are *n* static objects and *m* moving objects in the environment. We should predict the state and the covariance of the robot, stationary and moving objects (Line 2). The predicted states can be evaluated by:

(1) $${\hat{X}}_{k}^{-}=f_{k}\left({\hat{X}}_{k-1}^{+},u_{k}\right),$$

where $X_{k}\in {ℜ}^{\bar{n}}$ is the augmented state vector at time *t*_k_ with size of $\bar{n}=3+2n+4m$ and an initial condition $X_{0}$, and $u_{k}$ is the control inputs to the system at time *t*_k_. Note that ${\hat{X}}_{k}^{-}$ is the estimate of $X_{k}$ before the measurement $z_{k}$ is taken into account, and ${\hat{X}}_{k}^{+}$ is the estimate of $X_{k}$, after the measurement $z_{k}$ is taken into account. Moreover, *f_k_* is the kinematic model of the robot and objects [38]:

(2) $$f_{k}=\left[\begin{array}{c} x_{k}^{r}+\Omega_{k}^{r}\Delta t\;\cos\left(\phi_{k}^{r}+\Omega_{k}^{d}\Delta t\right)\\ y_{k}^{r}+\Omega_{k}^{r}\Delta t\;\sin\left(\phi_{k}^{r}+\Omega_{k}^{d}\Delta t\right)\\ \phi_{k}^{r}+\Omega_{k}^{d}\Delta t\\ X_{k}^{s}\\ T\;X_{k}^{d}+w_{k}^{d} \end{array}\right],$$

by defining $\Omega_{k}^{r}\equiv \left(\omega_{k}^{R}+\omega_{k}^{L}\right)\;r/2$ and $\Omega_{k}^{d}\equiv \left(\omega_{k}^{R}-\omega_{k}^{L}\right)\;r/D$, where *r* is the active wheels’ radius, *D* is the distance between the wheels, and Δ*t* is the sample time of the discrete fusion process. The variables $\omega_{k}^{R}$ and $\omega_{k}^{L}$ stand for the angular velocity of the right and the left wheels, respectively. Also, matrix *T* is the transition matrix for the sampling period Δ*t*, and $w_{k}^{d}$ is a zero mean Gaussian process noise with covariance matrix *Q_d_*. The transition matrix and the covariance matrix can be obtained by:

(3) $$T=\left[\begin{array}{cccc} 1 & 0 & \Delta t & 0\\ 0 & 1 & 0 & \Delta t\\ 0 & 0 & 1 & 0\\ 0 & 0 & 0 & 1 \end{array}\right],$$

and

(4) $$Q_{d}=\sigma_{Q}^{2}\left[\begin{array}{cccc} \Delta t^{4}/4 & 0 & \Delta t^{3}/2 & 0\\ 0 & \Delta t^{4}/4 & 0 & \Delta t^{3}/2\\ \Delta t^{3}/2 & 0 & \Delta t^{2} & 0\\ 0 & \Delta t^{3}/2 & 0 & \Delta t^{2} \end{array}\right],$$

where *σ*_Q_ is the standard deviation of the process noise.The covariance can be propagated by:

(5) $$P_{k}^{-}={\bar{F}}_{k}P_{k-1}^{+}{\bar{F}}_{k}^{T}+{\bar{G}}_{k},$$

where ${\bar{F}}_{k}$ stands for the Jacobian of $f:{ℜ}^{\bar{n}}\to {ℜ}^{\bar{n}}$ with respect to the state vector ${\hat{X}}_{k}^{+}$ at time *t_k_*:

(6) $${\bar{F}}_{k}=\left[\begin{array}{ccc} {\left(F_{k}\right)}_{3\times 3} & 0 & 0\\ 0 & I_{2n\times 2n} & 0\\ 0 & 0 & T_{4m\times 4m} \end{array}\right],$$

where *F_k_* can be obtained by:

(7) $$F_{k}=\left[\begin{array}{ccc} 1 & 0 & -\Omega_{k}^{r}\Delta t\;\sin\left(\phi_{k}^{r}+\Omega_{k}^{d}\Delta t\right)\\ 0 & 1 & \Omega_{k}^{r}\Delta t\;\cos\left(\phi_{k}^{r}+\Omega_{k}^{d}\Delta t\right)\\ 0 & 0 & 1 \end{array}\right],$$

and *I* is the identity matrix. Also, ${\bar{G}}_{k}$ can be obtained from:

(8) $${\bar{G}}_{k}=\;\left[\begin{array}{ccc} G_{k}Q_{v}G_{k}^{T} & 0 & 0\\ 0 & 0 & 0\\ 0 & 0 & Q_{d} \end{array}\right]\;,$$

where $G_{k}$ stands for the Jacobian of *f_k_* with respect to the control inputs $u_{k}$ at time *t_k_*:

(9) $$G_{k}=\left[\begin{array}{cc} \Delta t\;\cos\left(\phi_{k}^{r}+\Omega_{k}^{d}\Delta t\right) & -\Delta t^{2}\;\sin\left(\phi_{k}^{r}+\Omega_{k}^{d}\Delta t\right)\\ \Delta t\;\sin\left(\phi_{k}^{r}+\Omega_{k}^{d}\Delta t\right) & \Delta t^{2}\;\cos\left(\phi_{k}^{r}+\Omega_{k}^{d}\Delta t\right)\\ 0 & \Delta t \end{array}\right],$$

and the matrices *Q_v_* and *Q_d_* (4) are the error covariance matrices characterizing the noise in the robot model and dynamic objects, respectively. The predicted state can be projected into predicted measurements using the known nonlinear measurement equation:

(10) $${\hat{h}}_{k}^{i}=h_{k}^{i}\left({\hat{X}}_{k}^{-}\right),$$

and the innovation matrix:

(11) $$S_{i}=H_{i}P_{k}^{-}H_{i}^{T}+R_{i},$$

where $h:R^{\bar{n}}\to R^{\bar{m}}$ is known as vector measurement functions ($\bar{m}$ is the size of measurement vector):

(12) $$h_{k}^{i}=\left[\begin{array}{c} \sqrt{{\left(x_{q}^{\left(i\right)}-x_{k}^{v}\right)}^{2}+{\left(y_{q}^{\left(i\right)}-y_{k}^{v}\right)}^{2}}+v_{k}^{r}\\ \arctan\left(\frac{y_{q}^{\left(i\right)}-y_{k}^{v}}{x_{q}^{\left(i\right)}-x_{k}^{v}}\right)-\phi_{k}^{r}+v_{k}^{\theta } \end{array}\right],$$

where $x_{v}$ and $y_{v}$ are the position of the observation device at time step *k*. Index of *q* represents the *i*^th^ feature in the surrounding environment with the pose of $\left(x_{q}^{\left(i\right)},y_{q}^{\left(i\right)}\right)$ in the global coordinate X_G_-Y_G_. The position of *i*^th^ feature is indicated by $\left(r^{\left(i\right)},\theta^{\left(i\right)}\right)$ with respect to the observation device frame X_R_-Y_R_. The observation noise *ν_r_* and *ν_θ_* with the standard deviation *σ_r_* and *σ_θ_* are defined for the range and bearing noise, respectively. Also, *H*_i_ is the Jacobian of the measurement function *h*_i_ with respect to the state vector ${\hat{X}}_{k}^{-}$, and *R_i_* is the observation noise covariance matrix for the measurement *i*th assigned to the sensor. The measurement model of stationary objects can be obtained by linearization of (12):

(13) $$z_{k}=H_{k}^{s}X_{k}^{s}+v_{k},$$

where $H_{k}^{s}$ is the observation matrix. The measurement model of moving objects can be expressed as:

(14) $$z_{k}=H_{k}^{d}X_{k}^{d}+v_{k},$$

where $H_{k}^{d}=[\begin{array}{cc} H_{k}^{s} & 0_{2\times 2} \end{array}]$, and *ν* is zero-mean Gaussian noise with covariance *R*:

(15) $$R=\left[\begin{array}{cc} \sigma_{r}^{2} & 0\\ 0 & \sigma_{\theta }^{2} \end{array}\right].$$

Measurements are checked by individual compatibility search to find a potential match between measurements and tracks (Line 3). The best observations from observation *j*th to predicted measurement of track *i*th, are selected with smallest Mahalanobis distance to each track within a predefined validation gate, using normalized innovation squared:

(16) $$M_{ij}^{2}={\left(z_{j}-{\hat{h}}_{i}\right)}^{T}S_{i}^{-1}\left(z_{j}-{\hat{h}}_{i}\right),$$

then, the association matrix *J* is constituted:

(17) $$J=\{\begin{array}{c} \begin{array}{cc} 1 & if\;inlier\;\left(v<d_{1}\right) \end{array}\\ \begin{array}{cc} 0 & othervise \end{array} \end{array}.$$

The association matrix *J* describes the binary relationship of the measurements to the tracks without neglecting the ambiguous associations. The values of 1 and 0 in the matrix *J* indicate the measurement either is an inlier to a track or is not, respectively (Line 4). If the measurement *z* is an inlier to only one track, the associated track is updated by the measurement according to the association matrix *J* (Line 5). The state estimate can be updated by the normal Kalman filter equations (Line 6):

(18) $$\begin{array}{l} K_{k}=P_{k}^{-}H_{k}^{T}S_{k}^{-1},\\ {\hat{X}}_{k}^{+}={\hat{X}}_{k}^{-}+K_{k}\left(z_{k}-h\left({\hat{X}}_{k}^{-}\right)\right),\\ P_{k}^{+}=\left(I-K_{k}H_{k}\right)P_{k}^{-}, \end{array}$$

where *H*_k_ stands for the augmented Jacobian of all measurements and $h\left({\hat{X}}_{k}^{-}\right)$ transforms the features positions into the sensor coordinate. Computational efforts are reduced by finding the potential matching between estimated tracks and measurement features, before going to the RANSAC iterations. After updating the tracks which can be matched with only one measurement, some of the observations may not be associated with any track. When there are several observations around a track, connecting this track to actual observation needs a hard decision. So, when a track can be hypothetically matched with several tracks, the algorithm finds the best solution for deciding these observations using the RANSAC algorithm (Line 7.a). In the RANSAC section, *n* hypotheses are randomly generated from observation data and the estimated tracks. Initially, only the state vector is updated using these hypotheses and EKF formula. Then, all measurements are predicted to calculate the hypothesis consensus by counting measurements inside a threshold. At the end of this part, the hypothesis will be compared with the previous ones. If new hypothesis has larger consensus set than the maximum consensus of previous hypotheses, it will be stored as the best hypothesis. To ensure that a correct solution with probability *p* is found, the number of iterations *n_hyp_* is obtained by:

(19) $$n_{hyp}=\frac{\log\left(1-p\right)}{\log{\left(1-{\left(1-\varepsilon \right)}^{\bar{q}}\right)}^{}},$$

where $\bar{q}$ is the minimum number of data points necessary to find an estimation successfully, *ε* is the outliers’ ratio (percentage of outliers) in the data points. After determining the relationship between tracks and observations, the remaining tracks will be updated. (Line 7.b). If there is still another observation which needs a decision making, it could be found by increasing the value of the gating area and going to Line 7. In a crowded dynamic environment, this step helps the algorithm to make hard decisions smoothly. If a track is not updated for a specific time, it should be removed from the track list. If a measurement is an outlier to all existing tracks, it is added to the track list as a new track (Line 9). 

## 5. Results and Discussion

**Figure 5:**
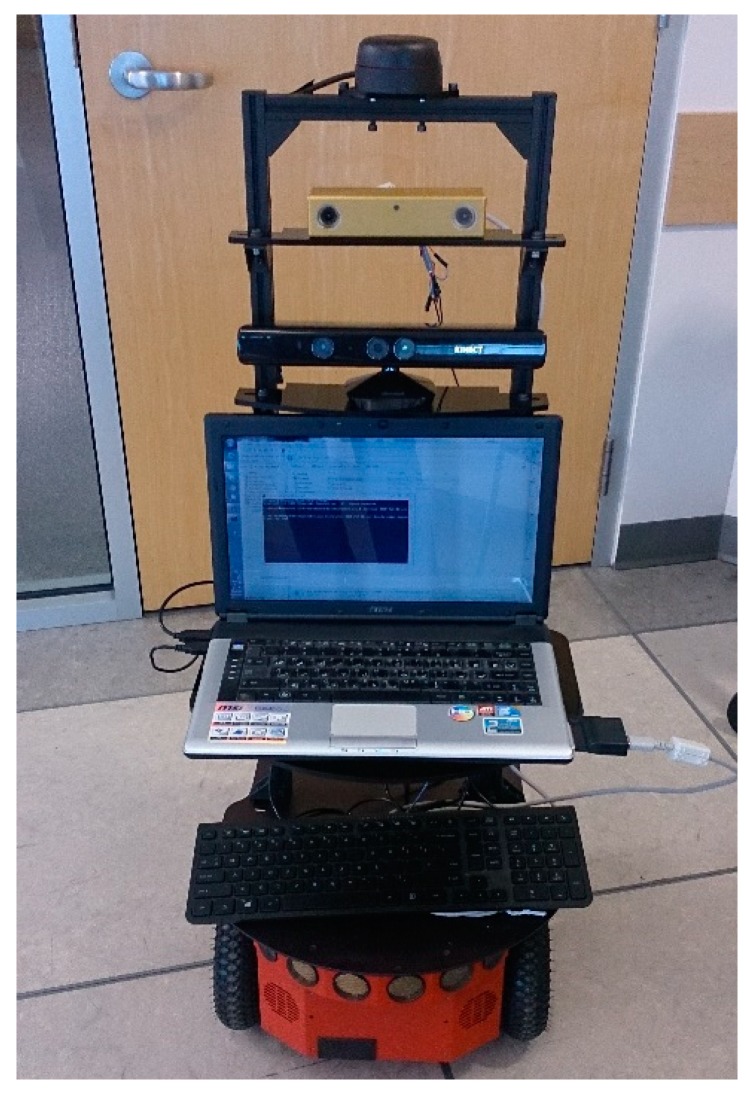
Pioneer P3-DX mobile robot with mounted sensors.

To evaluate the performance of the proposed ML-RANSAC SLAMMTT approach and trained R-CNN, a practical experiment was conducted. The experimental setup includes a Pioneer P3-DX (Figure 5) mobile robot in the MSE hallway of Simon Fraser University (Surrey campus). 

A 360 degrees RPLIDAR laser scanner is mounted on the top of the mobile robot. The RPLIDAR (a2) is a low cost 2D laser scanner with range of 8 to 16 meters, precision of 2 mm, scan frequency of 10 Hz, and resolution of 0.9 degree. The Bumblebee2 stereo vision system (model BB2-03S2C-38) with resolution of 648×488 and 3.8 mm focal length lenses (66-degree field of view) is mounted on the mobile robot, which has two Sony ICX424 cameras with each camera capable of taking a color image of high resolution at 48 fps (frames per second) rate. The baseline length is 12 cm. Although we could extract the depth data from stereo camera, we did not use them, because of saving computational efforts and memory usage. Images from either left or right camera can be used for mono vision application. LIDAR (RPLidar) and stereo vision are chosen to obtain dataset and validate the proposed algorithm, although different types of range finder sensors are installed on the mobile robot such as sonar and RGBD sensors. The results demonstrate that our approach can accurately and reliably estimate the robot pose, construct the map of static objects and keep track of moving objects. 

In this experiment, the Pioneer robot was moving with speeds of up to 20 cm/s in the dynamic environment. The position of objects was captured with respect to the robot by fusing the vision camera and the laser range finder with maximum distance of 8 meters. To increase the accuracy of the mapping, the detected doors with maximum distance of 3 meters were accepted to fuse with the vision data. The acceptable distance for people was set to 8 meters. The main objective of the LIDAR and vision data fusion is to geometrically align the output data of the sensors. Herein, we firstly detect objects via vision sensor, then we need to associate the detected patch in the camera with the corresponding data point obtained by the LiDAR sensor [43]. During all of the experiment, the observation noises were set to 0.3 m and 3 degrees for range and bearing, respectively. The update frequency was 7 scans per second for the laser range finder. If the estimator has no observation from features for a certain period of time (which was chosen 5 seconds in this experiment), the track will be removed. The robot navigated through the hallway while the position of the robot was being calculated by the IMU sensor, whereby the robot observed the surrounding environment by vision and laser sensors.

Along the navigation of the mobile robot through an indoor environment, the detection of doors in the robot route and their location are required. A pre-trained CNN network was used to be fine-tuned for door detection in our environment. The center point of the doors was calculated from the bounding box in the images. A calibration was required to find the center location of doors from the laser data. The total amount of images for training CIFAR-10 dataset was 60000, 32×32 color images in 10 classes [44]. Then, this pre-trained CNN is fine-tuned for door detection using 244 training images. For the training process, a GPU with NVIDIA GTX 745 consisting of 384 CUDA cores (with compute capability 5.0) was used in the experiment whereas the computer was equipped with core i7 3.4Ghz and 12G RAM. The learning rate was set to 0.001 at the beginning of the training. This initial value was used by the network training algorithm throughout the whole training process unless we wanted to change this value after certain number of epochs by multiplying with a coefficient. Choosing a large learning rate at the beginning of the training and gradually reducing this value during optimization, results in reducing the time of training whereas more accurate learning will be postponed to the end of the training. For the transition learning, we selected a smaller value, because most learning had already been completed. The initial learning rate is reduced each 8 epochs during the training process. Since we are using fine-tuning and transfer learning, the number of epochs is chosen 40. The momentum parameter is set to 0.9, which helps to accelerate the SGD algorithm in the relevant direction and reduces oscillations. The fine-tuning process took about 10 minutes. The training error was closed to zero after 16 epochs, and the test error was also stable. 

**Figure 6:**
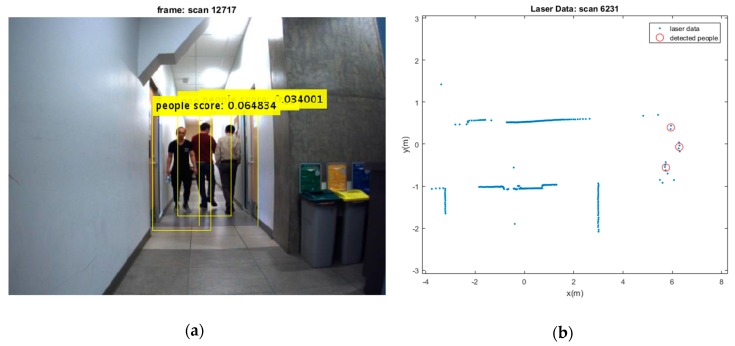
(**a**) Three detected moving people; (**b**) Their extracted depth from laser sensor.

**Figure 7:**
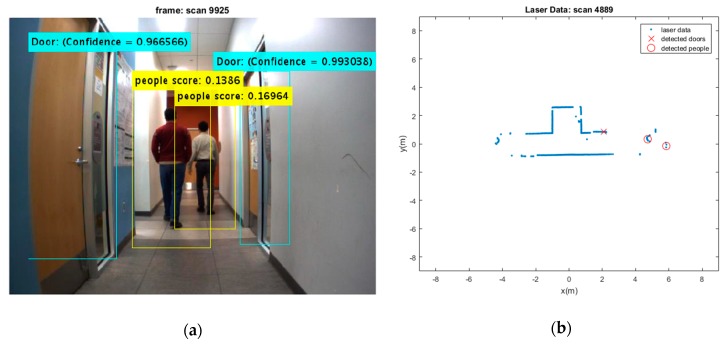
(**a**) Two detected moving people and detected doors; (**b**) Their extracted depth from laser sensor.

Two test results were chosen randomly from the dataset as shown in Figure 6 and Figure 7. The output results of the CNN for door detection are illustrated in Figure 7 by cyan bounding box. The output of the people detector is also shown by yellow bounding box in Figure 6 and Figure 7. Due to the space limit, the value of scores might not be shown in Figure 6. The current values are 0.064834, 0.11717, and 0.034001 from the left person to the right, respectively. The red cross mark denotes the center position of the doors in raw laser data, whereas the detected people are marked in red circle. There is no cross sign for the right door in Figure 7. This is caused by setting a parameter to limit door detection in distances less than 3 meters for increasing the accuracy in mapping of the environment. It should be noted that partial covering of an object might adversely affect the output of object detection, but it has still better performance as compared to the traditional algorithms.

The people and door detection results confirm the proposed object detection and classification from a practical visual scenario. It can be seen that the doors and people are detected successfully in Figure 7. It should be noted that many algorithms suffer from intermittent observation during the tracking process [45]. In a case that people detection loses the observation of people, the ML-RANSAC algorithm is useful to continue the tracking of people even by using the intermittent observation. Since the algorithm is developed for MTT, it allows to detect and track unlimited people in an image. 

**Figure 8:**
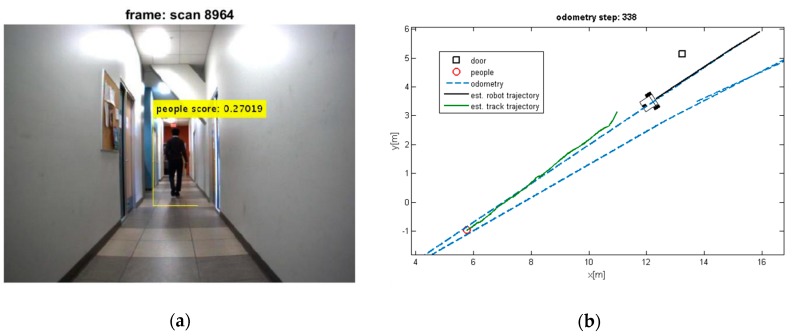
(**a**) Detected moving people; (**b**) Estimated trajectories of the robot and the tracks along with the estimated position of the doors.

**Figure 9:**
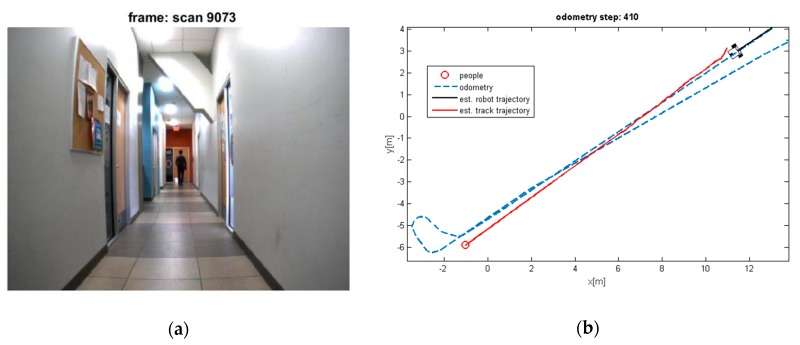
(**a**) Losing the detection of moving people; (**b**) Estimated trajectories of robot and tracks, whereas the proposed algorithm keeps tracking the moving object.

**Figure 10:**
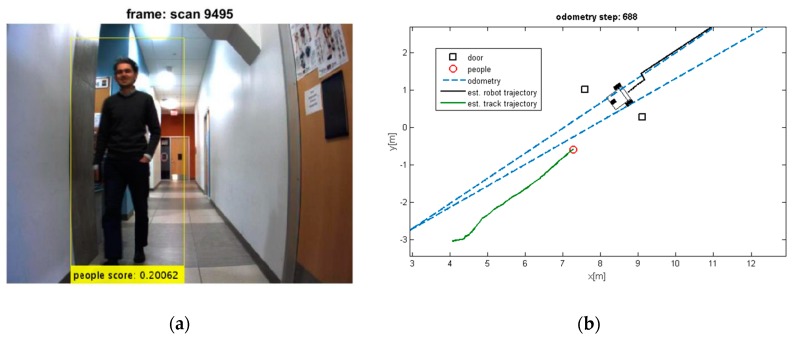
(**a**) Detected moving people; (**b**) Estimated trajectories of robot and tracks along with the estimated position of doors.

**Figure 11:**
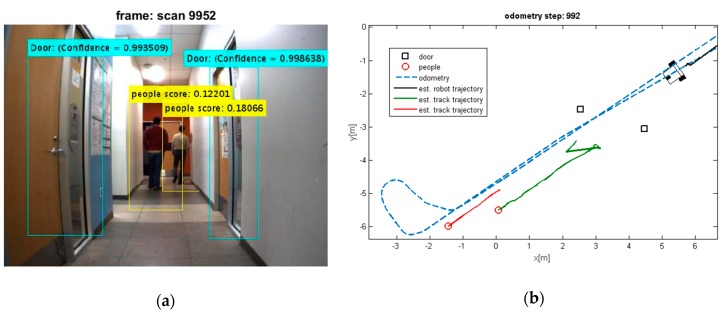
(**a**) Detected moving people and detected doors; (**b**) Estimated trajectories of robot and tracks along with the estimated position of doors.

The estimated trajectory of the moving objects along with the estimated position of robot and tracks are shown in Figure 8, Figure 9, Figure 10 and Figure 11. 

It can be seen that the moving objects are tracked correctly through the motion. The trajectories of the mobile robot and tracks are also shown in these figures. Although the dynamic objects (people) are moving away from robot (Figure 8 and Figure 9), sometimes they cannot be observed by sensors (Figure 9). In such cases, the estimator keeps tracking of the objects continuously while the mobile robot is moving and localizing itself. Thereby, it should be mentioned that the tracking algorithms will generally fail, if one of the two sample sets is removed or if one sample set tracks the wrong moving object, when proximity situation or occlusion situation takes place. In our experiment, that has happened in Figure 11. In this situation, at the first, one of tracks was associated with wrong observation because of occlusion situation, but after receiving new observation, it was able to track the correct person. The reason for losing a track usually comes from the intermittent observation from one of the features or more. It can be seen that using the proposed algorithm on the occlusion situation the system kept the constructing of the map and tracked the moving objects reliably. Thus, the multilevel modeling of RANSAC algorithm increases the performance of the system. It should be noted that the people are coming from both sides (front and back) in our experiment, but our camera can see the front. We can easily add another camera in the opposite direction (to the backward) to handle moving objects coming from behind.

**Figure 12:**
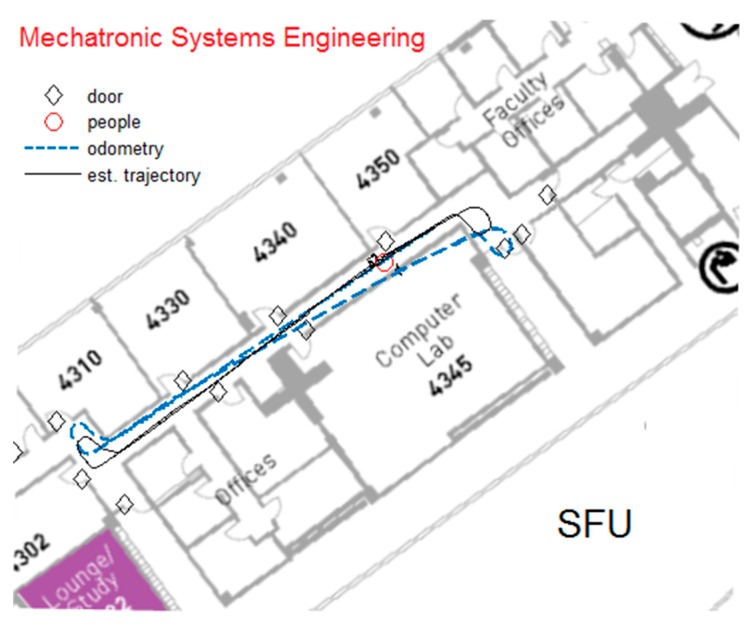
The estimated trajectory of the robot and the final estimated position of the doors in the architectural map of the environment.

The final estimated trajectory computed by the proposed algorithm along with the odometry path are shown in Figure 12. It can be seen that the estimated position of the doors is also compared with the ground truth information.

Herein, the continuous line indicates the estimated trajectory of the robot, whereas the odometry path is indicated by dashed line. The final map can be easily compared with the architectural map of the environment and the odometry data captured from IMU sensor. The odometry data show a drift in the path of the robot, which is corrected by applying the proposed method, while it can also detect and track the moving objects. It should be noted that if there is no object in the environment, the algorithm uses odometry data to move. If there are no doors and moving objects, the current trained network can also be trained for other objects in the environment. In such situations, we expect the proposed algorithm performs even better than other algorithms. As a prototype, we applied our algorithm to the current environment. The robot navigates in the experimental environment, which is 6 by 26 meters. However, it can be applied to a larger environment as well. The proposed algorithm can be implemented in real-time by using GPUs and FPGAs. For our future studies, we will employ a computer with GPU to extend the current prototype in real-time.

**Figure 13:**
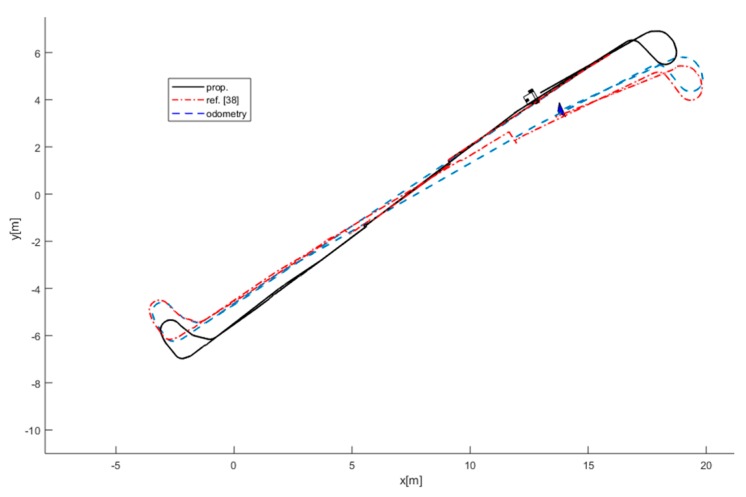
Comparison between the proposed method and described method in [38].

To provide a comparative assessment of performance of the proposed method in terms of accuracy in localization, mapping, and tracking, the estimated trajectory of the robot and the final map of the environment are compared with [38] and the result is shown in Figure 13. The described method in [38] employed only laser data for observation and traditional methods for object detection and tracking. Additionally, due to failing in the detection of the doors using traditional methods in the environment, it can be seen that the trajectory of the robot is not accurate and is disposed to the odometry path. Consequently, error in localization of the robot results in wrong tracking of the moving objects in the environment.

In addition, a qualitative comparison among the SLAM algorithms for dynamic environment is provided in Table 1. The main sensors for SLAM algorithms are laser and camera in this comparison. Indeed, the laser and vision data fusion facilitates robust feature detection and alleviates the computational cost in comparison to high computational techniques such as vision only algorithms. It can be seen that most of the algorithms are prone to error in pure dynamic environments. Additionally, the assumption of tracking only one target in [9,10,32,33], makes those impossible to be implemented in real dynamic environments. The algorithms of [8,10,11,12,32,36] are also locally applicable and require high computational effort and memory in large environments. The classical object detection methods are applied for object detection in these algorithms, which are not suitable for real environments and are prone to errors in unstructured dynamic environments. In comparison to available algorithms for dynamic environments, the proposed algorithm provides a robust data association using ML-RANSAC, is stable in a pure dynamic environment, supports MTT, is not limited to local mapping, carefully updates a track in proximity and occlusion situations, and provides a semantic segmentation for both static and dynamic objects by applying deep learning method for object detection.

## 6. Conclusions

We have presented a case whereby machine learning could be embedded within SLAM to address dynamic environments. In such scenarios, we require an algorithm for perception and object detection to solve the SLAM problem. In this paper, a novel algorithm has been proposed for robot autonomous navigation in dynamic environment. A deep learning algorithm has been applied via R-CNN to detect doors and people. The training procedure of R-CNN has been explained in detail. The proposed algorithm has resolved two main problems in dynamic environments: (i) object detection and classification and (ii) data association in the presence of multi moving objects. Experiments have been performed to validate our algorithm for door detection and people detection while doing SLAM and MTT. Experimental studies have verified that the proposed algorithm successfully tracks an unknown number of randomly placed moving objects. For future works, this algorithm can be applied to other types of SLAM, such as grid-based SLAM and graph-based SLAM, since the main ideas of the ML-RANSAC algorithm are: First, applying RANSAC method for data association in proximity and occlusion situations or in situations that we need a hard decision making. Second, updating states in a hierarchical approach to make hard decisions gradually and smoothly. The key point is to implement the method along with SLAM to handle the MTT and data association in a dynamic environment. Performing a hybrid solution on SLAM problem in dynamic environment might lead to better results, e.g., combining feature-based method for moving objects and an occupancy grid approach to represent fully static map. The ML-RANSAC framework can be extended to nonlinear systems without linearization by using the appropriate nonlinear filters, such as unscented Kalman filter and particle filter. Additionally, the filtering steps could be modified to model unknown target tracking using, for instance, the IMM filter.

## Figures and Tables

**Table 1:** A qualification comparison among the Simultaneous Localization and Mapping (SLAM) algorithms for dynamic environment.

Ref.	Sensors	Data Association Method	Pure Dynamic Environment	Comp. Cost	MTT	Local Mapping	Conflict Situation	Semantic Segmentation
[32]	Laser	GNN	NO	Middle	No	Yes	No	No
[8]	Laser	MHT	NO	High	Yes	Yes	No	No
[33]	Mono-camera	GNN	No	Very high	No	No	No	No
[9]	Stereo-camera	GNN	No	High	No	No	No	No
[10]	Laser	MHT	NO	High	No	Yes	No	No
[12]	Laser, stereo	GNN	NO	High	Yes	Yes	No	No
[11]	Laser, camera	MHT	NO	High	Yes	Yes	No	No
[36]	Laser, camera, radar	MHT	NO	High	Yes	Yes	No	No
[38]	Laser	RANSAC	Yes	Middle	Yes	No	No	No
Prop.	Laser, camera	RANSAC	Yes	High	Yes	No	Yes	Yes
